# Co_x_CrFeNiTi High-Entropy Alloys Prepared via Mechanical Alloying and Spark Plasma Sintering for Magnetron Sputtering Coatings

**DOI:** 10.3390/ma16196386

**Published:** 2023-09-24

**Authors:** Ciprian Alexandru Manea, Laura Elena Geambazu, Dorinel Tălpeanu, Virgil Marinescu, Gabriela Beatrice Sbârcea, Delia Pătroi, Radu Mihail Udrea, Magdalena Valentina Lungu, Mariana Lucaci

**Affiliations:** 1 National Institute for R&D in Electrical Engineering ICPE-CA Bucharest, Splaiul Unirii 313, 030138 Bucharest, Romania; ciprian.manea@icpe-ca.ro (C.A.M.); dorinel.talpeanu@icpe-ca.ro (D.T.); virgil.marinescu@icpe-ca.ro (V.M.); gabriela.sbarcea@icpe-ca.ro (G.B.S.); delia.patroi@icpe-ca.ro (D.P.); magdalena.lungu@icpe-ca.ro (M.V.L.); mariana.lucaci@icpe-ca.ro (M.L.); 2 Material Science and Engineering Faculty, University Politehnica Bucharest, Splaiul Independentei 313, 060042 Bucharest, Romania; 3 APEL LASER.SRL, Str. Vanatorilor 25, 077135 Mogosoaia, Romania; radu.udrea@apellaser.ro

**Keywords:** high-entropy alloy, mechanical alloying, spark plasma sintering, magnetron sputtering

## Abstract

The main objective of this study was to develop a high-entropy alloy (HEA) derived from the Co_x_CrFeNiTi HEA system (x = 0.5, 1) for protective coatings using the magnetron sputtering method. In order to produce the high-entropy alloy targets required for the magnetron sputtering process, mechanically alloyed metallic powders were consolidated via spark plasma sintering (SPS). The microstructural analysis results of the HEA mixture presented morphology changes after 30 h of alloying, with the particles presenting uniform polygonal shapes and dimensions. Subsequently, 316L stainless steel (SS) specimens were coated via magnetron sputtering, comparing their composition with that of the sputtering targets used for deposition to establish stoichiometry. Microstructural analyses of the SPSed HEAs revealed no defects and indicated a uniform elemental distribution across the surface. Furthermore, the CoCrFeNiTi equiatomic alloy exhibited a nearly stoichiometric composition, both in the coating and the sputtering target. The XRD analysis results indicated that amorphous coatings were obtained for both Co_0.5_CrFeNiTi and the CoCrFeNiTi HEA, and nanoindentation tests indicated that the CoCrFeNiTi HEA coating presented a hardness of 596 ± 22 HV, compared to the 570 ± 19 HV measured for Co_0.5_CrFeNiTi, suggesting an improved wear resistance.

## 1. Introduction

High-entropy alloys (HEAs), also known as multi-principal element alloys (MPEAs), represent a class of new design approach materials discovered by J.W. Yeh et al. [1] and B. Cantor et al. [2]. These alloy systems are composed of at least five principal elements, with chemical compositions varying between atomic percentages of 5 and 35, distributed in equal or near-equal amounts [1]. The HEA design concept relies on four core effects, with configurational entropy playing a key role in promoting the formation of solid solution phases over the intermetallic or complex phases [3].

HEAs exhibit superior properties when compared with the traditional alloys, including improved ductility, enhanced hardness, a good temperature stability, an anti-radiation ability, and improved oxidation, corrosion and wear resistance [4].

The advantages of using high-entropy alloys are mainly represented by the possibility of developing materials with tailored properties, which are determined by the chemical composition of the mixture, resulting in a major improvement of the mechanical and chemical properties in comparison with the classic alloys.

High-entropy alloys are known for their specific effects such as high entropy, slow diffusion, a severe distortion of the crystalline network and the cocktail effect, which are not found in other alloy systems. This phenomenon is called the “core effect” [5,6] having its origin in the complex interactions that occur between the constituent elements of high-entropy alloys.

The properties of high-entropy alloys depend mainly on the chemical composition of the materials, as well as their microstructure, electronic configuration and other characteristics. The advantage of the cocktail effect, specific to alloys with high entropy, is represented by the ability to obtain a final material with superior characteristics, acquired from the constituent elements [7].

The use of coatings made from materials with an improved resistance to wear helps to extend the lifespan of equipment that is affected by the destructive effects of premature or excessive wear. In terms of costs, wear leads to an increase in cost and maintenance intervals and, implicitly, to a decrease in productivity. HEA coatings also have an economic aspect due to the decrease in the production cost, having a major impact on the environment regarding the use of raw materials in comparison with its use as a bulk material [8].

The processing method used for the experimentation in this work was the powder metallurgy route, specifically mechanical alloying and spark plasma sintering. The choice of mechanical alloying offers the advantage of obtaining a higher level of homogeneity in the final alloyed powder material [9]. The mechanical alloying method is also preferred when aiming to prevent segregations which could potentially alter the mechanical properties, and when the constitutive elements are immiscible in a liquid state or exhibit significant differences in their melting temperatures.

As a consolidation method, SPS was selected for this research due to its fast processing times, high level of process control, high degree of densification and low oxidation, in comparison with the classic method of cold pressing followed by furnace sintering. By using SPS technology, sputtering targets for the magnetron can be obtained in near-net shape and size, with minimal mechanical machining required [10].

In addition to HEAs’ elemental compositions and the chosen processing route, the deposition technique also influences the properties of the final coatings. In recent experiments involving HEA synthesized coatings, the most used deposition technologies include electro-deposition, magnetron sputtering, electrochemical vapour deposition, laser cladding, arc coating, thermal spraying, and plasma transfer arc coating [11,12,13,14]. Among these methods, magnetron sputtering offers some advantages for deposition of HEA coatings in form of thin films due to its precise control over film chemistry, microstructure, and physical and mechanical properties [15]. Furthermore, due to the low energy of the particles bombarding the substrate, the deposition process is carried out at lower temperatures with no thermal effects on the substrate [16]. This results in enhanced atom mobility on the substrate’s surface and an improved microstructure of the film at lower substrate temperatures [17].

According to the literature [18], the high-entropy alloys from the face-centred cubic (FCC) single-phase CoCrFeNi system exhibit an increased tensile strength, but also a good ductility, even at low temperatures. The research presented in [18] was also conducted to explore the influence of titanium on the CoCrFeNi alloy. These studies have revealed a high hardness (~9.3 GPa) and compressive strength (~2.4 GPa), with primary FCC phases and secondary phases rich in Cr and Fe, resulting in an improved balance between resistance and toughness.

In the attempt at creating HEA metal coatings with superior properties, component elements such as cobalt, chromium, iron, nickel and titanium were selected. These elements are already used on an industrial scale as alloying elements, forming the basis for the production of cutting tools and alloys used in the production of equipment and component parts that work in chemically and mechanically aggressive environments, where properties such as wear, corrosion or fatigue resistance are essential [19].

For this study, two high-entropy alloys from the Co_x_CrFeNiTi HEA system were produced. Initially, in line with the cocktail effect, where each element contributes equally in order to obtain the superior properties of the final alloy, a CoCrFeNiTi HEA with an equiatomic composition was prepared.

For economic reasons, due to the high price of cobalt powder, a composition with a low cobalt content, specifically Co_0.5_CrFeNiTi, was selected for a comparative study. This choice was made to observe the influence of the cobalt concentration on the properties of the final alloys from the Co_x_CrFeNiTi system and to reduce the overall alloy production cost.

## 2. Materials and Methods

High-purity metallic powders of Co (<40 µm, purity > 99.5%), Cr (<100 µm, purity > 99+%), Fe (<85 µm, purity > 99.9%), Ni (<10 µm, purity > 99+%) and Ti (150 ÷ 250 µm, purity > 99.5%) were alloyed in a planetary ball mill (Fritsch Pulverisette 5/2 Classic line, Idar-Oberstein, Germany) using stainless steel vials and balls at a speed of 250 rpm for 30 h, with a 10:1 ball to powder ratio (BPR) and N heptane as a process control agent (PCA). The role of the PCA was to prevent the oxidation or contamination of the powders in order to improve the material’s behaviour during the consecutive welding and re-welding of the mechanical alloying process, and to reduce the tendency of powders to adhere to the walls of the vials or to the balls. The elaboration of the Co_x_CrFeNiTi HEA with a variable cobalt content (x = 0.5; 1) was achieved through mechanical alloying. This method was chosen due to several factors, including its low processing cost, and its ability to achieve a high degree of alloying and homogeneity throughout the entire mass of the alloy.

Two HEA compositions were produced, specifically CoCrFeNiTi with equal atomic ratios of each element, 20% in this case, and low-cobalt-content Co_0.5_CrFeNiTi, with 11.2 at% Co and 22.2 at% for the other elements.

The mechanically alloyed powders were sieved to determine the average particle size, using vibrating sieving equipment (Fritsch Analysette 3 Spartan, Germany) and sieves with openings of 160 µm, 140 µm, 125 µm, 100 µm, 80 µm, 63 µm, 56 µm, 45 µm, 32 µm and 20 µm. The percentage remaining on each sieve from the total amount used to determine the particle size fraction was used to calculate the average particle size according to Equation (1).

(1) $$\mathrm{Average}\;\mathrm{particle}\;\mathrm{size}=\frac{100}{\sum \frac{m}{\mathrm{Dm}}}$$

where

m—Percentage amount remaining on each sieve;
Dm—Average diameter between two consecutive sieves.

The processing of the HEA powders was carried out in a vacuum with spark plasma sintering (SPS) equipment (HP D25 FCT Systeme GmbH, Effelder-Rauenstein, Germany), using a high-density graphite die with an inner diameter of 20.8 mm, lined with a graphite foil with a thickness of 0.4 mm, and 20 mm diameter pushing rods to fabricate the SPSed samples, with a diameter × thickness of 20 mm × 4–5 mm. To avoid a temperature gradient, the die assembly was covered with 5 mm of thick graphite felt.

In order to establish the final sintering parameters for the magnetron sputtering targets, in correlation with other experimentations from the literature [20,21] and adjusted for the present research, several tests were carried out by varying the sintering temperature from 800 °C to 975 °C, with the other parameters remaining constant: a pressure of 50 MPa, a heating/cooling rate of 50 °C/min and a dwell time of 5 min.

For the microstructural surface analyses, the sintered HEA samples were sandblasted to remove the graphite foil, metallographically prepared by polishing with abrasive paper and rinsed with high-purity ethyl alcohol (96%).

The Vickers hardness of the SPS-consolidated mechanically alloyed HEAs was measured with the XMO195 series FM700 Micro hardness tester equipment, at a room temperature of 23 °C.

The apparent density of the sintered samples was measured using a hydrostatic balance (XS204 METTLER TOLEDO, Greifensee, Switzerland) using Archimedes’ principle. The liquid medium used was ethanol at 96% purity, at a temperature of 21 °C. For each sample, 3 sets of measurements were made (in air and immersed in liquid), and then the average value and the standard deviation were calculated. The densification degree was calculated using Equation (2):

(2) $$\mathrm{Densification}\;\mathrm{degree}=\frac{\rho_{a}}{\rho_{t}}*100$$

where

ρ_a_—Apparent density;
ρ_t_—Theoretical density (7.15 g/cm^3^ for the CoCrFeNiTi equiatomic HEA and 6.99 g/cm^3^ for the Co_0.5_CrFeNiTi low-cobalt-content HEA).

The theoretical densities were calculated according to the mass percentages and densities of each constituent element, according to Equation (3).

(3) $$\rho_{t}=\frac{100}{\sum \frac{m_{e}}{\rho_{e}}}$$

where

m_e_—element wt%;
ρ_e_—element density.

After analysing the results of the hardness and density tests, and according to the dimensional specifications of the magnetron sputtering equipment, the sputtering targets were sintered using a die with an inner diameter of 40.8 mm, lined with a graphite foil with a thickness of 0.4 mm and 40 mm diameter pushing rods. The processing parameters are presented in Table 1.

The deposition trials were performed using magnetron sputtering equipment developed by Apel Laser, Romania. The system includes a magnetron sputtering source (AJA International, Scituate, MA, USA), a high-voltage power supply (PREVAC, Rogów, Poland), a quartz microbalance (PREVAC, Poland) and a rotating substrate holder. The deposition substrate, 316L SS, was chosen, with a length × width × thickness of 42 mm × 25 mm × 2 mm. During the thin film deposition, the SS substrate was rotated at 15 rpm, but not heated. The coatings were produced in a DC regime in an Ar atmosphere, with the deposition parameters that are presented in Table 2.

Initially, a pre-sputtering step of 5 min was performed in order to clean the sputtering target surface. Afterward, the shutter was opened, and the deposition process was initiated. The deposition lasted for 20,000 s, which is equivalent to 5.5 h. The estimated thickness of the deposited layer was 1000 nm, with the deposition rate being monitored using the quartz microbalance.

The HEA powders, sintered specimens and coatings were analysed using scanning electron microscopy (SEM) and energy-dispersive X-ray spectroscopy (EDS) with a Carl Zeiss FESEM-FIB Workstation, Auriga, Germany, equipped with the X-MaxN energy-dispersive X-ray spectroscopy (EDS) module from Oxford Instruments equipment.

To identify the crystalline phases, X-Ray diffraction (XRD) analysis was performed with a D8 Discover instrument from Bruker, Germany, equipped with Cu primary radiation (λ = 1.54060 Å), a Göebel mirror and a 1D LynxEye detector on the secondary side equipment. The diffractograms were recorded with an angular increment of 0.04° at a scanning speed of 1 step/s. The analysis of the coatings was performed via grazing incidence at a low angle of 1°, in order to exclude the crystalline structure of the substrate. The crystallographic phases were identified using the ICDD PDF 2 2022 Release database.

The mechanical characterization consisted of performing microscratch and nanoindentation tests to measure the Vickers hardness (HV) and Young’s modulus (E_IT_). The nanoindentation tests were performed for the magnetron sputtering HEA coatings and the uncoated SS substrate by making five measurements on each sample with a mechanical characterization equipment Micro-Combi Tester (MCT^2^), equipped with a nanoindentation module (NHT^2^), and a Berkovich diamond indenter with a *ν*_i_ = 0.07 Poisson’s coefficient and an E_i_ = 1140 GPa elasticity modulus (CSM Instruments, Peseux, Switzerland). The nanoindentation test parameters for the magnetron sputtering HEA coated samples are the following: a maximum indentation depth of 95 nm (maximum indentation load of 2 mN, linear force loading), an indenter approach speed of 4 μm/min, a loading/unloading speed of 2 µm/min and a data acquisition frequency of 10 Hz.

The nanoindentation tests parameters for the uncoated SS substrate are the following: a maximum indentation load of 400 mN (non-linear force loading (quadratic loading, where the indentation force (F) is F = k xt2, where k represents the rate of increase in the indentation force (mN/min), and t is the indentation time (min)) with an indenter approach speed of 2 μm/min, a maximum loading/unloading time of 30 s, a maximum load duration time of 10 s and a data acquisition frequency of 10 Hz.

Microscratch tests were performed for the magnetron sputtering HEA coated samples using the same testing equipment, a Micro-Combi Tester (CSM Instruments, Peuseux, Switzerland), equipped with a microscratch module (MCTX) with a 100 µm radius diamond Rockwell tip, an acoustic emission sensor (EA), a penetration depth detection sensor and a tangential force and friction coefficient detection sensor. By performing the microscratch tests, the critical forces (Lc) were determined, i.e., the lowest loads at which visible defects appear in the investigated coated samples, using the recorded curves and the images captured with the Panorama option of the data acquisition software. The microscratch test parameters are the following: a progressive linear scratching force from 0.03 N to 15 N, a constant scratching speed of 5 mm/min, a scratch length of 5 mm, a loading speed of 15 N/min and a scanning contact force of 30 mN.

The values of the mechanical parameters were determined for the *ν* = 0.3 Poisson’s coefficient using the Oliver and Pharr calculation method [22]. The results were obtained after calculating the arithmetic mean value of the measurements and the standard deviation.

The samples were tested at an ambient room temperature of 23 ± 2 °C and a relative air humidity of 35 ± 3%.

## 3. Results and Discussion

**Figure 1:**
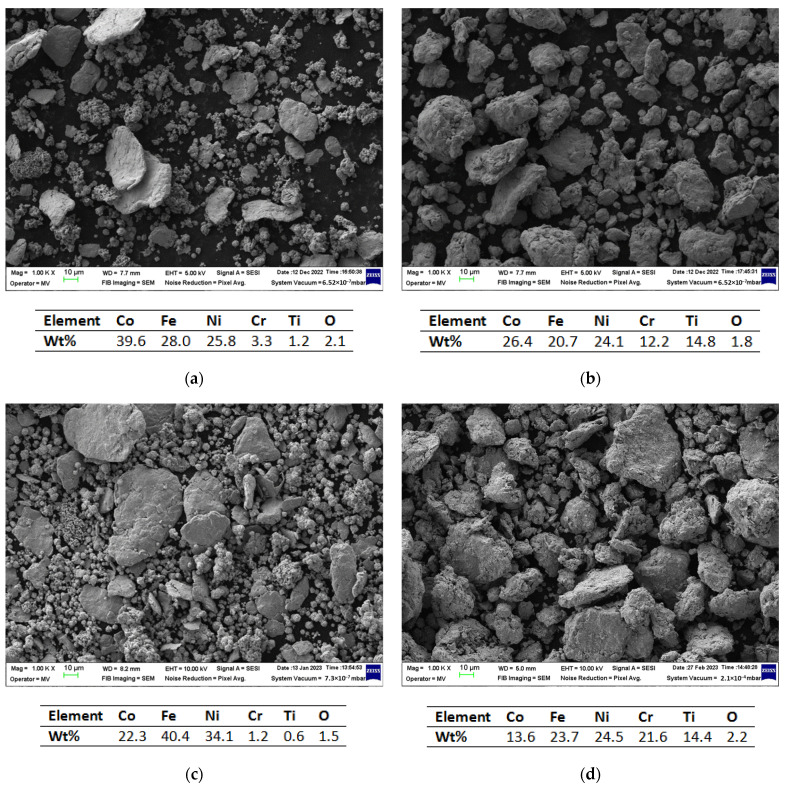
SEM and EDS comparative analysis results for the CoCrFeNiTi equiatomic (**a**) homogenized sample and (**b**) after 30 h of mechanical alloying, and for the Co_0.5_CrFeNiTi (**c**) homogenized sample and (**d**) after 30 h of mechanical alloying.

Figure 1 presents the results of the SEM and EDS analyses performed on the Co_x_CrFeNiTi system alloys. In Figure 1a, the homogenized sample of the CoCrFeNiTi equiatomic mixture is presented, while Figure 1b presents the results after 30 h of mechanical alloying of the equiatomic mixture. Figure 1c presents the mixed sample of the Co_0.5_CrFeNiTi mixture and Figure 1d presents the analysis results after 30 h of mechanical alloying of the Co_0.5_CrFeNiTi mixture.

The SEM images of the mixed samples reveal the dimensional differences between the elementary powder particles as well as their particular geometric shapes (Figure 1a,c), while the analysis of the alloyed powder indicate a shape and dimensional uniformity of the HEA powders (Figure 1b,d). For the alloyed metallic powders, it can be observed that the particles agglomerated due to continuous welding and fracturing, indicating the completion of the mechanical alloying process.

The EDS analysis confirms the presence of all constituent elements in the analysed area without contamination from other elements. Due to the boiling point of the PCA being 98.38 °C, no traces were identified in the chemical analyses, being under the detection limit of the EDS analyse. In a paper published by H. Kotan et al. [23] for a Zr/Y doped CoCrFeNi HEA produced via mechanical alloying, with Dodecane (0.7 wt%) as the PCA, and consolidated via the SPS process into a graphite die, it was reported that the presence of C in a Cr_7_C_3_ phase was detected via TEM microstructural analyses. The C contamination resulted from the PCA utilized in the mechanical alloying process and further annealing, but also it was observed that the HEA metallic powders reacted with the C from the sintering die set during the SPS process. Carbon contamination was also observed in the case of the AlCoCrFeNi HEA that was mechanically alloyed with methanol and toluene as the PCA [24], where Cr_7_C_3_ phase formation was reported upon annealing. A complete FCC phase was identified after mechanically alloying the mixture for 15 h, using toluene as the PCA; C traces were observed from the XRD analyses but could not be confirmed due to the detection limits. Carbide phase formation was observed more clearly after the mechanically activated annealing [24]. By correlating this work’s results with the wider literature, the possibility of C contamination of the CoCrFeNiTi HEA studied is not excluded.

The presence of oxygen was detected in the elemental mixture of metallic powders, with the measured concentration reaching a maximum value of 2.2 wt%.

**Figure 2:**
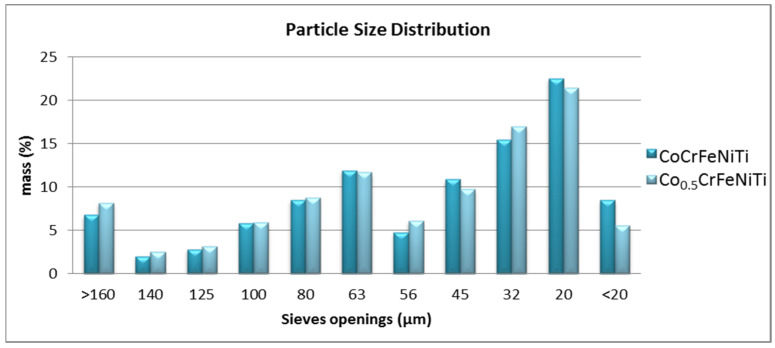
Graphical comparison of the particle size distribution for the CoCrFeNiTi equiatomic and Co_0.5_CrFeNiTi high-entropy alloys.

The particle size and morphology could influence the porosity of the sintered sample and the compaction behaviour during the SPS process, but also the final density of the sintered sample, thus leading to the particle size distribution analyses of the alloyed metallic powders. The microstructural analyses revealed a reduction in particle size for both high-entropy alloys. The results that were assessed by sieving the HEA powders are presented in Figure 2.

From the graphical representation of the particle size distribution, it could be observed that the studied alloys exhibit similar results, with the highest material fractions being in the range of 20–45 µm in diameter.

**Figure 3:**
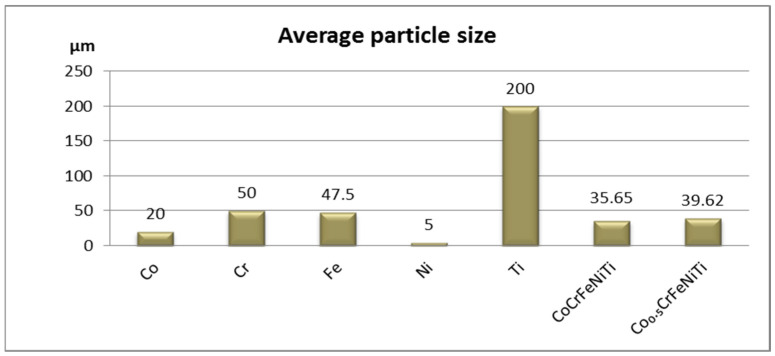
Average particle size comparison between CoCrFeNiTi, Co_0.5_CrFeNiTi and elemental metallic powders.

Figure 3 presents a comparison of the average particle sizes between CoCrFeNiTi, the Co_0.5_CrFeNiTi HEAs and the elemental metallic powder materials, showcasing the average particle sizes. The CoCrFeNiTi HEA powder had a calculated value of 35.65 μm, while the Co_0.5_CrFeNiTi HEA had a calculated value of 39.62 μm, which was higher than that of the equiatomic alloy. This increase could be attributed to the higher concentration of ductile elements, such as iron, nickel and titanium, in particular. In contrast to the average particle size of the Ti powder, which was approximately 200 μm, there was a substantial reduction in the final powder size, confirming the results obtained from the SEM analyses.

**Figure 4:**
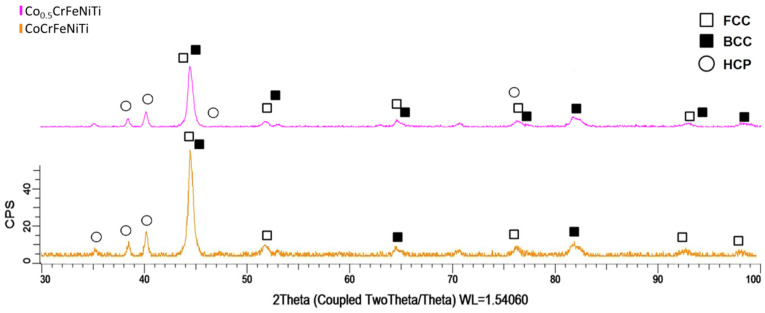
XRD comparative analyses between CoCrFeNiTi and the Co_0.5_CrFeNiTi HEA that were mechanically alloyed for 30 h.

In order to validate the valence electron concentration (VEC) theory [25], according to which the studied materials will have a mixture of FCC and body-centred cubic (BCC) phases, X-ray diffractometry analyses were carried out on samples collected after 30 h of mechanical alloying for the CoCrFeNiTi equiatomic and Co_0.5_CrFeNiTi HEA (Figure 4).

The XRD analysis results presented in Figure 4, for both the CoCrFeNiTi and Co_0.5_CrFeNiTi HEA powders, revealed the presence of the FCC, BCC and minor hexagonal (HCP) phases, which align with the VEC calculations that yielded a value of 7.4. HCP phases generally indicate a low mechanical resistance, but in both cases, the coexistence of the BCC phase in the crystalline structure induces an optimal ductility and resistance ratio, as mentioned in the literature [26,27].

For the CoCrFeNiTi equiatomic HEA powder, the highest peak intensity was identified at the 2θ angular position of 44°, with a majority of FCC and BCC phases. For the Co_0.5_CrFeNiTi HEA powder, the highest peak intensity was identified at the 2θ angular position of 44.5°, preferentially oriented on the (111) plan, featuring a Ni-rich FCC phase. Similar to the equiatomic alloy, a mixture of FCC and BCC phases was observed.

After the mechanical alloying process and the characterization of metallic powders of the studied alloys, the next step involved vacuum consolidation using the SPS technique.

The sintering temperature was increased gradually from 800 °C to 900 °C, then 950 °C and, finally, to 975 °C, to assess its influence on the physical characteristics of the sintered HEA samples. After the metallographic preparation of the sintered samples from both compositions, the Vickers hardness and apparent density were measured and the densification degree was calculated. The resulting values are presented in Table 3.

The results of the hardness measuring indicated an increase in both HEAs with the increase in the sintering temperature, and simultaneously, an increase in the apparent density, reaching values close to the theoretical densities. Microcracks caused by the indenter were observed for the samples sintered at 975 °C.

P. Kratochvil and F. Prusa [20] produced a near-equiatomic CoCrFeNiTi HEA via mechanical alloying for a total of 10 h and 30 min at 400 RPM, and compacted the metallic powder via SPS at 48 MPa and 1000 °C for 1 min, followed by rapid cooling for 9 min, resulting in a value of 757 ± 18 HV 30. As the authors observed, a good value of compressing strength was obtained (1340 MPa) with no plastic deformation, which indicated a brittle behaviour. The results are comparable with the results obtained after sintering the material at 975 °C. A similar brittle behaviour was observed by Shun et al. [28], where, with increase in the Ti content (0–11 at%), the plasticity was affected for a CoCrFeNiTi_x_ HEA obtained via arc melting. The authors observed that, for x = 0.5, a hardness value of 515 HV 1 was measured, which is lower than the hardness values obtained in this work.

The increased hardness-induced embrittlement could potentially result in cracks or even failure during subsequent mechanical processing of the sputtering targets. After analysing the results of the preliminary tests, the selected SPS parameters for producing sputtering targets were the following: a sintering temperature of 950 °C, a pressure of 50 MPa, a heating rate/cooling of 50 °C/min and a dwell time of 10 min.

**Figure 5:**
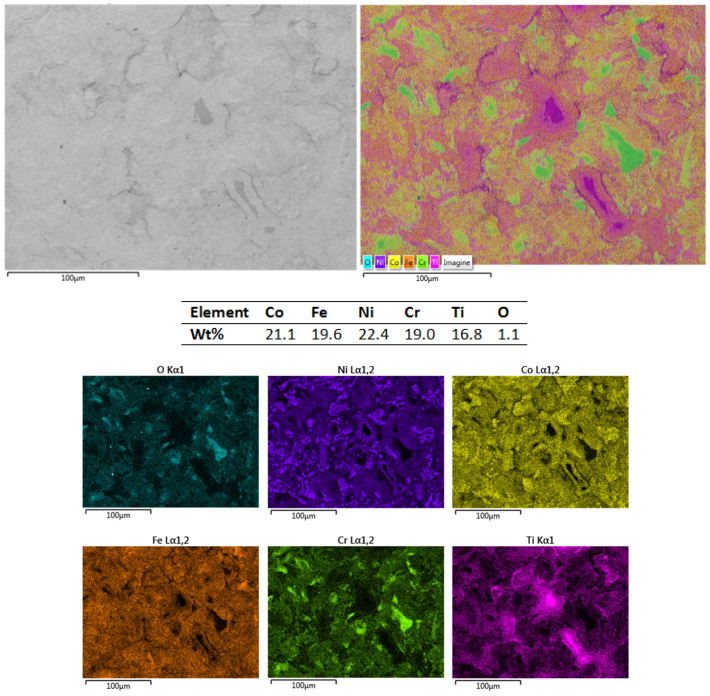
SEM, EDS and mapping analysis results for the CoCrFeNiTi equiatomic HEA sputtering target.

The CoCrFeNiTi equiatomic HEA and the Co_0.5_CrFeNiTi low-Co-content HEA sintered sputtering targets underwent SEM and EDS analyses to observe their structure and chemical composition, followed by mapping analyses to assess the elemental distribution. Figure 5 presents the results for the CoCrFeNiTi equiatomic HEA sputtering target.

As can be observed, the elemental composition was confirmed via both the EDS and mapping analyses. A superficial oxidation was detected, but there was no contamination with other elements in the composition of the processed materials, as revealed via the EDS analyses. A uniform distribution and high homogeneity were observed, and although there were areas with an abundance of Ni, it was distributed throughout the mass, forming compounds with Co, Cr and Ti. No major surface defects were observed that could affect the integrity of the obtained target or the subsequent deposition process.

**Figure 6:**
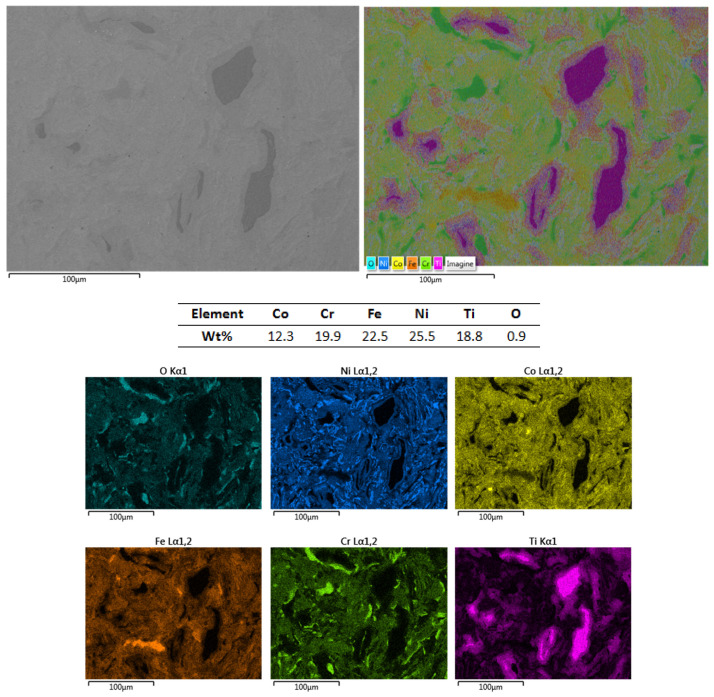
SEM, EDS and mapping analysis results for the Co_0.5_CrFeNiTi low-cobalt-content HEA sputtering target.

Figure 6 presents the results of the SEM, EDS and mapping analyses for the Co_0.5_CrFeNiTi low-cobalt-content HEA sputtering target.

In this case, a zonal abundance of Ti and Cr was observed. The superficial oxidation of Cr was identified in the mapping analyses and, according to the EDS analyses, resulted in a 1.8 wt% of oxygen on the selected analysed area. As indicated in the SEM microstructural analyses, no visible defects that could affect the integrity of the sample were detected. The presence of fine dendrites confirms that the developed material belongs to the high-entropy alloys domain [29,30,31]. This type of structure was also present in the CoCrFeNiTi equiatomic HEA and the Co_0.5_CrFeNiTi low-cobalt-content HEA. Although zonal abundances of elements were identified, they were uniformly distributed across the analysed area, with no segregations observed.

**Figure 7:**
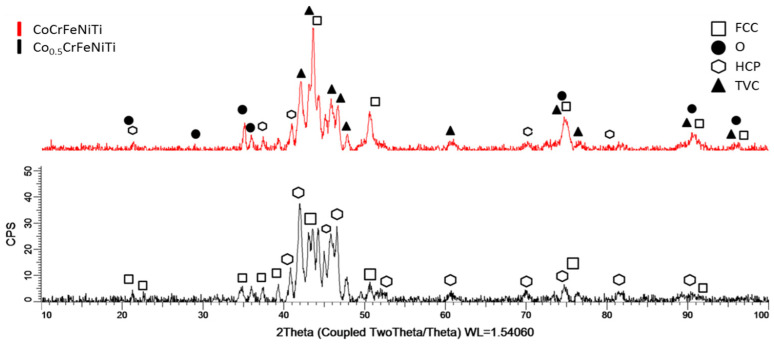
Comparative XRD analyses performed for the elaborated sputtering targets.

Figure 7 presents the results of the XRD analyses for both sputtering targets obtained from the high-entropy alloys studied in this paper.

In the CoCrFeNiTi equiatomic HEA sample, major phases of tetragonal (TVC) and FCC were identified for the Cr-Ni and Fe-Ni compounds, occurring at the 2θ angular position of 44°. These phases were also identified at the 2θ angular positions of 42°, 50° and 73°, and as minor phases at the 2θ angular positions of 48°, 61°, 77° and 90°. Additionally, minor HCP phases were identified at positions 21°, 38°, 41°, 70° and 80° for the Ti compounds. The presence of HCP phases could be attributed to an incomplete reaction of the Ti particles, resulting in compounds with Ni. Furthermore, similar to the SPSed samples with 20 mm diameters, compounds of Ni, Cr and their oxides identified as orthorhombic phases were observed, indicating the reproducibility of the sputtering target fabrication process. In this case, the VEC calculations were confirmed by the presence of the tetragonal and FCC phases, indicating favourable ductility and strength properties in the studied material. The presence of the TVC phase indicates the possibility for forming both the FCC and BCC phases, thereby confirming the VEC theoretical calculations.

In the Co_0.5_CrFeNiTi low-cobalt-content HEA sample, it was observed that, as the cobalt content decreased, and, implicitly, the content of the other elements, especially titanium, increased, mostly HCP and FCC phases were identified. In this case, TVC phases were not identified. Titanium primarily formed compounds with Ni and Fe, resulting in HCP phases, while the FCC phases were attributed to the presence of Cr compounds. Additionally, a similarity in the angular positions where crystallographic phases were identified was observed.

**Figure 8:**
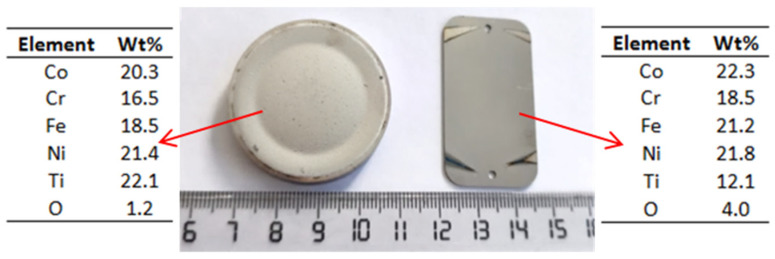
Macroscopic images and EDS results of the CoCrFeNiTi equiatomic HEA sputtering target and the coating obtained from using this sputtering target.

The first attempts to produce HEA coatings on a 316L SS substrate via the magnetron sputtering deposition technique were made using the CoCrFeNiTi equiatomic HEA target and the parameters described in Section 2. The outcome was a nearly-equiatomic HEA coating, as illustrated in Figure 8.

**Figure 9:**
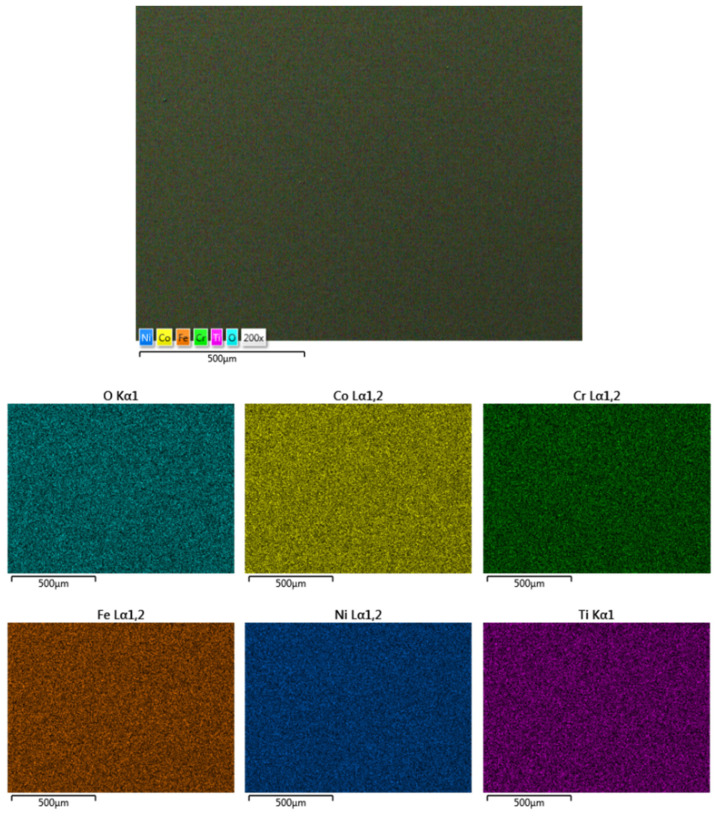
Results of mapping analyses for coating made from the equiatomic HEA sputtering target.

As it can be observed at the macroscopic level, material was extracted/ripped off from the HEA sputtering target and subsequently deposited onto the SS substrate. Upon closer examination, a minor delamination of the deposited layer was observed, requiring adjustments to the deposition parameters, as well as the preparation of the substrate prior to the deposition process. The target chemical analysis was performed on the magnetron sputtering extraction area. In order to verify the composition of the deposited layer from the HEA sputtering target, SEM, EDS and mapping analyses were carried out, with the results displayed in Figure 9.

**Figure 10:**
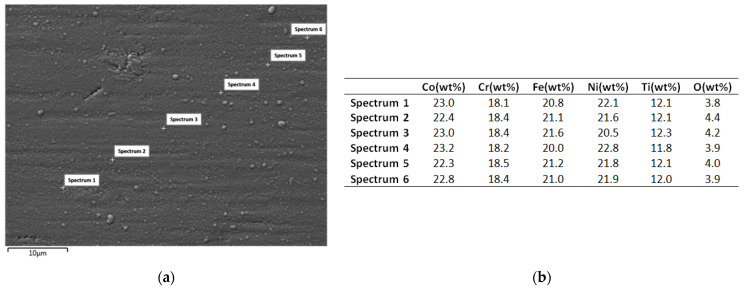
Results of SEM (**a**) and EDS (**b**) analyses for the coating made from the CoCrFeNiTi equiatomic HEA sputtering target.

As is noticeable from the mapping analysis results, the coating surface obtained from the equiatomic high-entropy alloy CoCrFeNiTi sputtering target exhibited a uniform distribution of the constituent elements, with a high homogeneity across the entire analysed area. The presence of oxygen was also detected, and to determine its concentration, EDS analyses were carried out; the results are presented in Figure 10.

The EDS analysis was carried out at multiple points to assess the repeatability and consistency of the layer deposited using the magnetron sputtering coating technique (Figure 10a). At all selected points, the chemical composition of the deposition target was confirmed. From a stoichiometric point of view, the most noticeable difference was observed for titanium, showing an approximately 5 wt% variation compared to the target composition prior to deposition. The difference could be attributed to the larger particle size of titanium compared to the other elements, as well as its distribution. When comparing the EDS analyses of the CoCrFeNiTi HEA sputtering target before and after the deposition process, it was observed that titanium exhibited a lower deposition rate than the other elements; its concentration increased in the sputtering area of the target while the other elements were transferred to the substrate, and a preferential extraction of the constituent elements was produced.

In each analysed area, the presence of approximately 4 wt% of oxygen was detected (Figure 10b), indicating a superficial oxidation of the coating. This phenomenon may indicate the passivation effect of the coating. From the SEM surface analysis of the coating, a continuous deposition was observed, characterized by limited defects or voids and minimal topographical differences.

**Figure 11:**
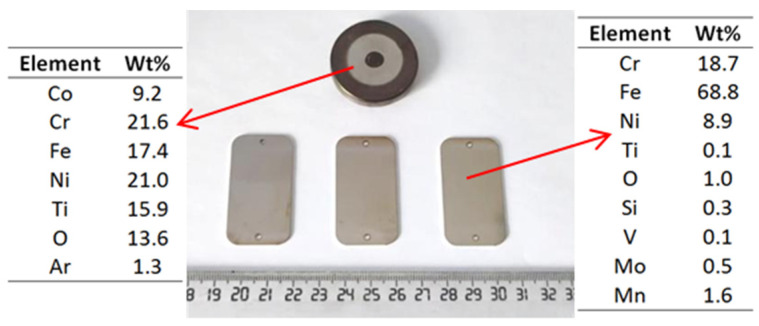
Macroscopic image and EDS results of the Co_0.5_CrFeNiTi low-cobalt-content HEA sputtering target and the coatings obtained from this target.

Taking into consideration the results obtained for the use of the CoCrFeNiTi equiatomic HEA sputtering target, the deposition experiments for the Co_0.5_CrFeNiTi low-cobalt-content HEA target were correlated in order to prevent a possible delamination that may affect the functional properties of the coatings. The macroscopic results for these coatings and for the used target after magnetron deposition are presented in Figure 11.

Compared to the equiatomic HEA magnetron target presented macroscopically in Figure 8, the low-cobalt-content HEA target shows a high degree of oxidation on the surface.

**Figure 12:**
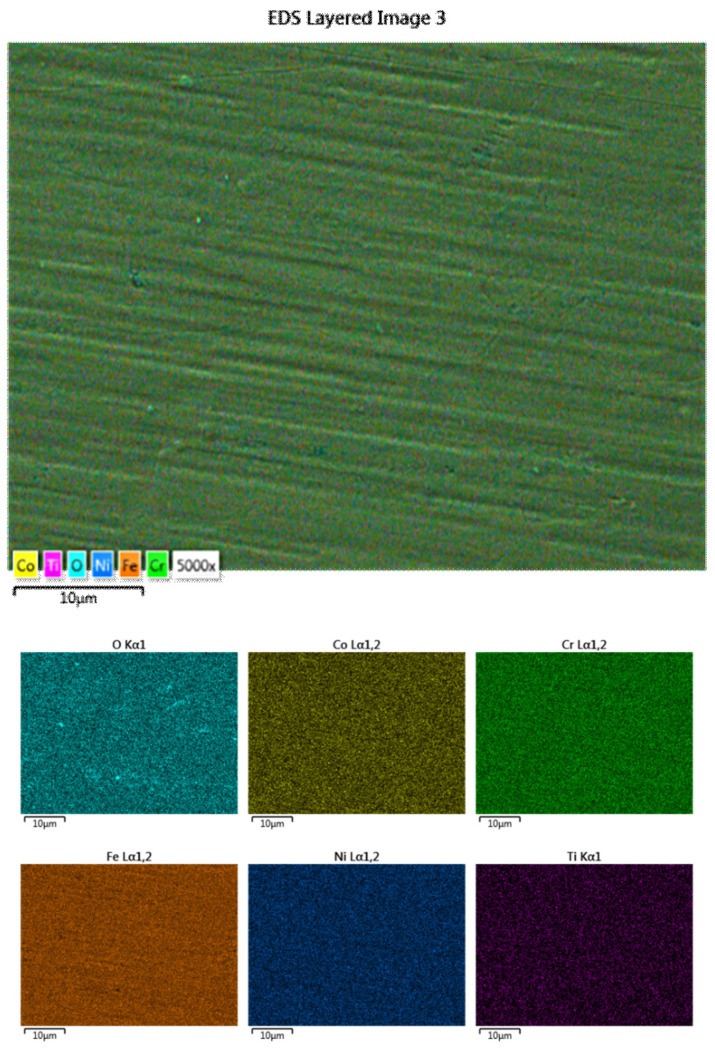
Results of mapping analyses for the coating made from the Co_0.5_CrFeNiTi low-cobalt-content HEA sputtering target.

The initial analyses carried out on these coatings involved SEM and EDS in order to identify the constitutive elements and assess the quality of the coating. The analysis results are presented in Figure 12.

The SEM surface analysis of the coated sample revealed a continuous deposition without any cracks or other defects. In the EDS analysis of the selected area, a compositional disproportionality in comparison with the sputtering target was observed, where cobalt was not identified in the coating’s chemical composition.

In order to observe the distribution of the elements, the surface mapping analysis was carried out. Although cobalt was not observed as a constituent element from the EDS spectrum, its presence was identified as traces in the mapping analysis. As can be seen, the elements are uniformly distributed on the surface, with oxygen also being present. For this analysis, only the constituent elements were taken into consideration.

**Figure 13:**
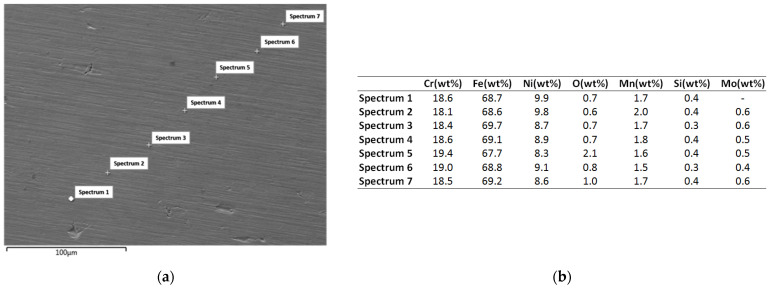
SEM (**a**) and EDS (**b**) analyses results for the coating obtained from the Co_0.5_CrFeNiTi low-cobalt-content HEA sputtering target.

In Figure 13, the SEM and EDS analysis results are presented. As for the coating obtained from the equiatomic high-entropy alloy, multiple areas were analysed in order to observe the coating’s continuity and homogeneity throughout the surface.

When analysing the surface for the coating obtained from the Co_0.5_CrFeNiTi low-cobalt-content HEA sputtering target, it was observed that, besides cobalt, which was not observed previously as a constituent element from the EDS spectrum, titanium was not identified throughout the analysed spectra, but traces were identified in the mapping analyses presented in Figure 12. There was an observed continuity in the chemical composition, with low oxidation (max. 2.1 wt% for the analysed area), but the coating contained elements from the SS substrate that was used for the deposition.

The magnetron coating from the Co_0.5_CrFeNiTi HEA sputtering target could no longer be considered as a high-entropy alloy because the necessary criteria were not met, namely that the proportion of each element should be between atomic percentages of 5 and 35 [6]. In addition to the constituent elements of the target, other elements such as Mn, Si and Mo were observed in small quantities, with these belonging to the 316L SS substrate on which the deposition was made. Their appearance may be due to the diffusion from the surface of the coating.

**Figure 14:**
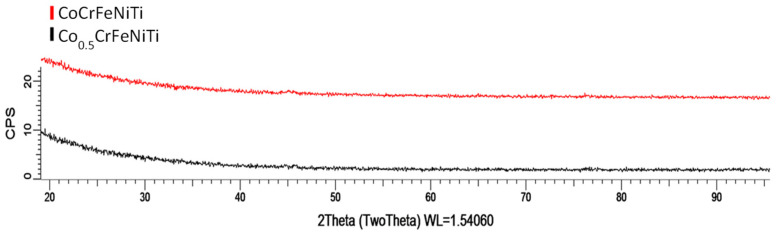
GI-XRD comparative analyses results for the CoCrFeNiTi equiatomic HEA and Co_0.5_CrFeNiTi low-cobalt-content HEA coatings deposited via magnetron sputtering.

In order to determine the crystallinity of the coatings and the phases that had formed, GI-XRD analyses were performed on the CoCrFeNiTi equiatomic HEA and Co_0.5_CrFeNiTi low-cobalt-content HEA coatings deposited via magnetron sputtering. The comparative results are presented in Figure 14.

When analysing the comparative XRD results of the HEA coatings deposited via magnetron sputtering, it became evident that no phases had formed, with the coatings having an amorphous structure. When comparing the results with the recent literature, M. Poliakov et al. [32] presented the possibility of depositing CoCrFeNiTi_x_ thin films via magnetron sputtering deposition on Si/SiO_2_, with targets produced via the hot pressing of the powder mixture. XRD analyses presented the occurrence of an amorphous coating, which persisted after performing an annealing treatment at 400 °C, and as for the case presented in this manuscript, a smooth surface with a uniform distribution of elements was obtained.

The results obtained in the current experimentation indicated that the process parameters were not adequate for the development of crystalline structures, highlighting the need for further experimentation.

**Figure 15:**
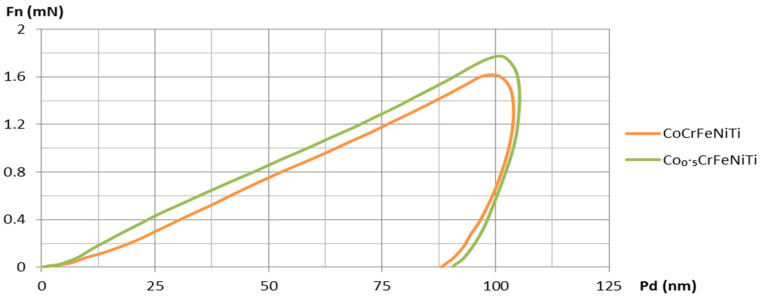
The loading/unloading vs. displacement curves comparative of the CoCrFeNiTi equiatomic HEA and Co_0.5_CrFeNiTi low-cobalt-content HEA coatings deposited via magnetron sputtering.

**Figure 16:**
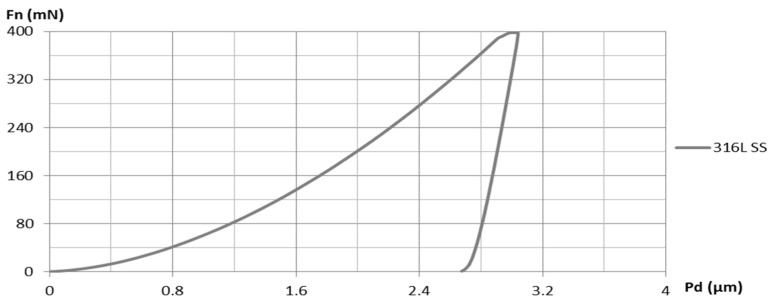
The loading/unloading vs. displacement curve for the 316L stainless steel substrate.

The coated samples and the SS substrate were mechanically characterized via nanoindentation in order to measure the Vickers hardness (HV) and Young’s modulus (E_IT_). The loading/unloading vs. displacement curves are presented in Figure 15 and Figure 16.

The instrumented nanoindentation measurements that are presented in Table 4 revealed superior mechanical characteristics for both HEA coatings compared to the SS substrate. The indentation hardness (H_IT_) of the HEA coatings was approximately 2.8 to 2.9 times higher than that of the SS substrate, indicating a greater resistance to irreversible (plastic) deformation as well as reversible (elastic) deformation [33]. The CoCrFeNiTi equiatomic HEA coatings exhibited higher H_IT_ values than the Co_0.5_CrFeNiTi HEA coatings, suggesting a better wear resistance. Furthermore, a similar trend was observed for the Vickers hardness.

The indentation modulus (E_IT_), which provides a reasonably accurate approximation of the Young’s modulus [34], was about 2 to 2.4 times higher for the HEA coatings compared to that of the SS substrate. Higher E_IT_ values for the HEA coatings indicate a better resistance to reversible deformation [33] when compared to the SS substrate.

The H_IT_/E_IT_ ratio, also referred to as true hardness [33], is higher for both HEA coatings (0.0244–0.0279) compared to the SS substrate (0.0200). A higher true hardness in the HEA coatings signifies an improved resistance to plastic deformation and enhanced wear resistance [33,35]. However, the low values of the H_IT_/E_IT_ ratio, which can be assimilated to an “elasticity index” [33], suggest that there is a tendency toward permanent deformation in both the HEA coatings and the SS substrate.

The H_IT_^3^/E_IT_^2^ ratio demonstrated that the Co_0.5_CrFeNiTi HEA coating exhibited a resistance to plastic deformation of 0.0048 GPa, which was approximately 1.3 times greater than that of the CoCrFeNiTi equiatomic HEA coating (0.0038 GPa) and 5.3 times greater than that of the SS substrate (0.0009 GPa). Nevertheless, the H_IT_^3^/E_IT_^2^ ratio of the CoCrFeNiTi equiatomic HEA coating (0.0038 GPa) was 4.2 times higher than that of the SS substrate (0.0009 GPa).

The above-mentioned findings demonstrate that the deposition of protective Co_x_CrFeNiTi (x = 0.5, 1) HEA coatings via magnetron sputtering has enhanced the hardness, elastic modulus, wear resistance and resistance to plastic deformation of the 316L SS substrate used in our study. Moreover, the mechanical properties of the magnetron sputtered Co_x_CrFeNiTi (x = 0.5, 1) HEA coatings, with a thickness of 1 µm, developed by us are comparable to data reported in the literature [35]. For instance, the CoCrFeMnNiTi_x_ (x = 0, 0.25, 0.5, 0.75 and 1.0 molar ratio) HEA coatings, with thicknesses of up to 900 µm and prepared using laser cladding, exhibited H_IT_ values ranging from 2.87 ± 0.48 GPa to 5.67 ± 0.99 GPa, Vickers hardness HV values between 348 ± 6 and 511 ± 5, E_IT_ values from 199 ± 6 GPa to 316 ± 1 GPa, H_IT_/E_IT_ ratios of 0.0144–0.0186, and the H_IT_^3^/E_IT_^2^ ratios of 0.00059–0.00196 [35]. The variations in mechanical properties can be attributed to the differences in the chemical composition of the developed HEA coatings.

**Figure 17:**
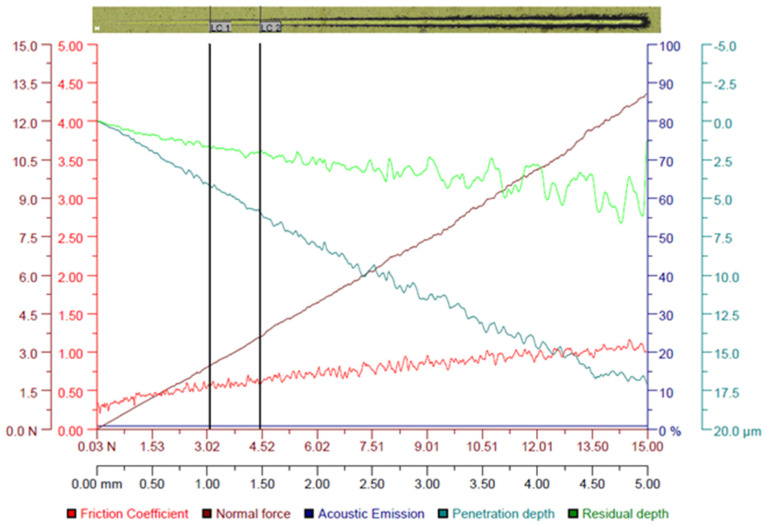
Variation of the friction coefficient, normal force, acoustic emission, penetration depth and residual depth along the scratch length for the CoCrFeNiTi HEA coating/steel (L_C1_ = 3.09 N, L_C2_ = 4.46 N). The top image shows the Panorama optical image (20× magnification and 25 µm scale bar) of the top surface of the sample after scratch testing.

**Figure 18:**
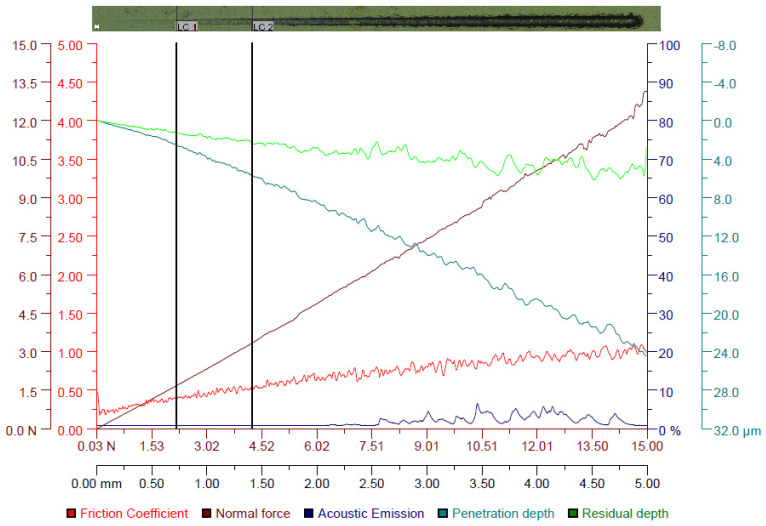
Variation of the friction coefficient, normal force, acoustic emission, penetration depth and residual depth along the scratch length for the Co_0.5_CrFeNiTi HEA coating/steel (L_C1_ = 2.19 N, L_C2_ = 4.26 N). The top image shows the Panorama optical image (20× magnification and 25 µm scale bar) of the top surface of the sample after scratch testing.

For both HEA coatings, it can be observed in Figure 17 and Figure 18 that the magnitudes of the friction coefficient, normal force (F_n_), penetration depth (P_d_) and residual depth (R_d_) increase as the scratch test progresses from 0.03 N to 15 N along the scratch length of 5 mm. Furthermore, constant acoustic emissions of 0.85% are noticed for the CoCrFeNiTi HEA coating along the scratch length of 5 mm, while the acoustic emissions of the Co_0.5_CrFeNiTi HEA coating were initially constant (0.89%) along the scratch length of up to 2.6 mm, then fluctuated up to 5% along the scratch length of 2.6–5 mm.

These findings indicate a better adhesion of the CoCrFeNiTi equiatomic HEA coating compared to that of the Co_0.5_CrFeNiTi HEA coating. The optical critical loads (L_C_), defined as L_C1_ (initial cracking of the HEA coatings with minor cracks localized at the scratch track edges) and L_C2_ (extensive cracking and failure in the HEA coatings with the delamination of the HEA coating from the steel substrate and exposing the steel substrate), were higher for the CoCrFeNiTi equiatomic HEA coating (L_C1_ = 3.09 N, L_C2_ = 4.46 N) compared to the Co_0.5_CrFeNiTi HEA coating (L_C1_ = 2.19 N, L_C2_ = 4.26 N), indicating a higher microscratch resistance. In the first portion of the scratch length, where the penetration depth (P_d_) of the Rockwell indenter was up to 1 µm, which corresponds to the thickness of the HEA coatings, the friction coefficient was in the range of 0.3–0.373 for the CoCrFeNiTi equiatomic HEA coating, and 0.3–0.472 for the Co_0.5_CrFeNiTi HEA coating. After that, until the end of the scratch length, the friction coefficient gradually increased up to one, due to the delamination of the HEA coatings. Accordingly, the higher friction coefficient can be attributed to the steel substrate. In addition, both HEA coatings exhibited fluctuations in the P_d_ plots along the scratch length of 5 mm, which can be attributed to the localized plastic (irreversible) deformation, which also indicates the fracture of the HEA coatings. However, the CoCrFeNiTi equiatomic HEA coating exhibited lower P_d_ and R_d_ values and higher relaxation than the Co_0.5_CrFeNiTi HEA coating, suggesting better wear resistance. Moreover, the microscratch results are in agreement with the nanoindentation results presented in this study.

## 4. Conclusions

As a result of the experiments carried out in this study, two compositions from the Co_x_CrFeNiTi HEA system (x = 0.5, 1) were successfully synthesized using 99+% purity raw metallic powders via mechanical alloying in a planetary ball mill.

The alloyed HEA powders have been analysed via scanning electron microscopy and compared with the mixed metallic powder in order to observe the morphological changes regarding the shape and dimension. The mixed HEA powder sample exhibited a dimensional difference between the elementary powder particles, along with distinct geometric shapes. In contrast, the analyses of the alloyed HEA powders indicated particles that were more uniform in both their shapes and dimensions. The EDS analysis confirmed the alloys’ composition and the lack of contaminants. Additionally, the comparative XRD analysis revealed the transition of the major phase from BCC to FCC, depending on the chemical composition. Furthermore, the presence of HCP was identified in both the equiatomic and low-Co-content HEAs.

In order to consolidate the alloyed metallic powders and establish suitable parameters, the spark plasma sintering (SPS) process was performed to achieve samples with a diameter × thickness of 20 mm × 4–5 mm, and magnetron sputtering targets with a diameter × thickness of 40 mm × 7 mm. The microscopic and chemical analyses of the SPSed HEAs revealed no defects, which confirmed the composition and indicated a uniform elemental distribution across the surface.

Using the magnetron sputtering process, coatings of CoCrFeNiTi equiatomic and Co_0.5_CrFeNiTi low-cobalt-content high-entropy alloys were deposited onto SS substrates. The XRD results revealed amorphous coatings from both HEA sputtering targets. However, for the Co_0.5_CrFeNiTi HEA, the chemical compositions and mapping analysis results indicated that traces of titanium and cobalt were present, resulting in the coating being ineligible to be classified as a HEA. In contrast, the coatings from the equiatomic HEA sputtering target exhibited a nearly stoichiometric composition with the sputtering target.

From the nanoindentation results, the CoCrFeNiTi equiatomic HEA coatings exhibited higher H_IT_ values when compared with the Co_0.5_CrFeNiTi HEA coatings, suggesting an improved wear resistance.

From the scratch test analyses, it resulted that the CoCrFeNiTi equiatomic HEA coating exhibited lower P_d_ and R_d_ values and higher relaxation when comparing the results with the Co_0.5_CrFeNiTi HEA coating, indicating an improved wear resistance, and confirming the nanoindentation results.

Future research will be focused on adjusting the magnetron sputtering parameters to produce coatings with crystalline structures. Moreover, a suitable thermal treatment of the amorphous HEA coatings highlighted in this study will be considered. Furthermore, the next goal is to apply protective HEA coatings that are optimized for superior properties onto different substrates using the obtained magnetron sputtering targets.

## Figures and Tables

**Table 1:** Spark plasma sintering process parameters for HEA magnetron sputtering targets.

Temperature (°C)	Pressure (MPa)	Heating Rate (°C/min)	Cooling Rate (°C/min)	Dwell Time (min)
950	50	50	50	10

**Table 2:** Magnetron sputtering parameters for high-entropy alloy depositions.

Gas (sccm)	Target–Substrate Distance (cm)	Calibration Pressure (Torr)	Calibration Power (W)	Deposition Rate (Å/s)	Deposition Power (W)
30	12	6.0 × 10^−3^	78	0.50	78

**Table 3:** The physical characteristics for the spark plasma sintered HEA samples.

No. Crt.	Chemical Composition	Sintering Temperature (°C)	Average Hardness Value HV ± std. dev.	Apparent Density ± std. dev. (g/cm^3^)	Densification ± std. dev. (%)
1.	CoCrFeNiTi	800	380.20 ± 38.09	5.958 ± 0.001	83.32 ± 0.024
2.		900	553.16 ± 23.88	6.845 ± 0.001	95.73 ± 0.021
3.		950	708.94 ± 94.42	7.100 ± 0.002	99.30 ± 0.037
4.		975	709.20 ± 4.64	7.135 ± 0.002	99.79 ± 0.037
5.	Co_0.5_CrFeNiTi	800	342.03 ± 2.65	5.881 ± 0.002	84.13 ± 0.025
6.		900	671.60 ± 38.39	6.877 ± 0.002	98.38 ± 0.029
7.		950	804.10 ± 28.18	6.973 ± 0.002	99.75 ± 0.028
8.		975	806.40 ± 16.03	6.975 ± 0.016	99.79 ± 0.239

**Table 4:** The mechanical characteristics of the Co_x_CrFeNiTi (x = 0.5, 1) HEA coatings and 316L SS substrate, determined using instrumented nanoindentation and the Oliver and Phar calculation method.

Sample	Indentation Hardness (H_IT_) ± std. dev. (GPa)	Vickers Hardness HV ± std. dev.	Indentation Modulus (E_IT_) ± dev. std. (GPa)	H_IT_/E_IT_	H_IT_^3^/E_IT_^2^ (GPa)
CoCrFeNiTi HEA coating	6.437 ± 0.235	596 ± 22	264 ± 14	0.0244	0.0038
Co_0.5_CrFeNiTi HEA coating	6.157 ± 0.201	570 ± 19	221 ± 14	0.0279	0.0048
316L SS substrate	2.183 ± 0.054	207 ± 4	109 ± 2	0.0200	0.0009

## Data Availability

Not applicable.
